# Acceptability and optimisation of resources to support antidepressant cessation: a qualitative think-aloud study with patients in Australian primary care

**DOI:** 10.3399/BJGP.2023.0269

**Published:** 2023-11-28

**Authors:** Suzanne McDonald, Katharine Wallis, Mark Horowitz, Esther Mann, Vilany Le, Maria Donald

**Affiliations:** General Practice Clinical Unit, Medical School, University of Queensland, Brisbane, Australia.; General Practice Clinical Unit, Medical School, University of Queensland, Brisbane, Australia.; Research and Development Department, North East London NHS Foundation Trust, London, UK.; University of Queensland, Brisbane, Australia.; University of Queensland, Brisbane, Australia.; General Practice Clinical Unit, Medical School, University of Queensland, Brisbane, Australia.

**Keywords:** antidepressive agent, deprescriptions, qualitative research

## Abstract

**Background:**

Stopping long-term (>12 months) antidepressant use can be difficult due to unpleasant withdrawal symptoms. Many people do not recognise withdrawal symptoms or understand how to minimise them while safely discontinuing antidepressants. To address the gaps, the authors developed the ‘Redressing long-term antidepressant use’ (RELEASE) resources, comprising a medicines information brochure, a decision aid, and drug- specific hyperbolic tapering protocols.

**Aim:**

To explore patients’ acceptability of the RELEASE resources to optimise their use and impact.

**Design and setting:**

A think-aloud interview study among adults with lived experience of long-term antidepressant use conducted in south-east Queensland, Australia, between November 2021 and June 2022.

**Method:**

Participants were purposively sampled from general practices and interviewed face-to-face or via videoconferencing. Participants verbalised their thoughts, impressions, and feelings while engaging with each resource. Interviews were analysed using a deductive coding framework, including codes related to acceptability and optimisation. Interviews were analysed in a series of four tranches, with iterative modifications made to resources after each tranche.

**Results:**

Participants (*n* = 14) reported the resources to be relevant, informative, motivational, and usable. Participants’ comments informed modifications, including changes to wording, content order, and layout. Several participants expressed frustration that they had not had these resources earlier, with one reporting the information could have been ‘life changing’. Many commented on the need for these resources to be widely available to both patients and doctors.

**Conclusion:**

The RELEASE resources were found to be acceptable, useful, and potentially life changing. The effectiveness of these consumer-informed resources in supporting safe cessation of long-term antidepressants is currently being tested in general practice.

## Introduction

Around half of people who try to stop using antidepressants experience unpleasant withdrawal symptoms, which can last for weeks or months and sometimes longer.^1^^,^^2^ Withdrawal symptoms are more common and last longer than previously understood. Symptoms can be disabling, and are frequently misconstrued as relapse by both patients and doctors resulting in ongoing and long-term (>12 months) use.^3^^–^^6^ Withdrawal symptoms can be minimised by hyperbolic tapering of antidepressant dose.^3^^,^^7^ Hyperbolic tapering describes a process of decreasing drug dose in progressively smaller decrements to give steady decrease in brain receptor occupancy. Slow tapering of drug dose is now recommended in clinical guidelines.^8^^,^^9^

Most antidepressants are prescribed in general practice, therefore, safe cessation needs to be supported in general practice to address increasing long-term antidepressant use. Guidance on tapering, and availability of drug mini doses for tapering, help to address the fear of relapse and to manage withdrawal symptoms, which have been identified as critical to successful antidepressant cessation.^4^ To this end, the authors developed three resources:

- a medicines information brochure;
- a decision aid; and
- drug-specific hyperbolic tapering protocols.

The medicines information brochure provides information about antidepressants and is designed to raise awareness and recognition of antidepressant adverse drug effects and withdrawal symptoms.

The decision aid is designed to support informed decision making about continuing or stopping antidepressant use.

The drug-specific tapering protocols provide step-by-step guidance for a slow tapering of antidepressant drug dose. The resources are being tested in the Redressing long-term antidepressant use (RELEASE) cluster randomised controlled trial in Australian general practice.

It is important that the design of these resources is informed by people with lived experience of long-term antidepressant use to ensure that they are acceptable and useful to adults who might need them. This article reports a think-aloud study among adults with lived experience of long-term antidepressant use to assess acceptability and to optimise the resources.

**Table:** How this fits in

It is not known how best to support patients to safely stop long-term (>12 months) antidepressant use when there is no clinical indication for continued use. The current study tested and optimised three patient resources designed to raise awareness and recognition of withdrawal symptoms and to provide step-by-step guidance for tapering drug dose to minimise withdrawal symptoms. Adults with lived experience of long-term antidepressant use reported that the resources were useful, acceptable, clear, comprehensible, and reassuring.

## Method

### Design

The think-aloud method involves asking participants to verbalise their thoughts, impressions and feelings while engaging with a resource to elicit perspectives about content, functionality and usability. This method has been used successfully to assess the acceptability and usability of a range of patient health resources.^10^^–^^13^

### Setting, participants and recruitment

A purposive sample of adults was recruited through four general practices from The University of Queensland GP Research Network in south-east Queensland, Australia between November 2021 and June 2022. Inclusion criteria were being aged >18 years and taking antidepressants (selective serotonin re-uptake inhibitors or serotonin and norepinephrine re-uptake inhibitors) for ≥12 months either currently or in the past 5 years. Participants were excluded if they were under the care of a psychiatrist; had severe depression; psychotic or obsessive-compulsive disorder; were taking antidepressants for reasons other than depression or anxiety, for example, pain; and/or were not able to read English.

Recruitment methods included advertisements displayed in the practice waiting area and verbal invitations from a GP during a consultation; the GP provided a handout containing information about the study, contact details for the research team and a QR code linked to a webform for an expression of interest. A researcher contacted participants, confirmed eligibility, sent the participant an information sheet via email and scheduled an interview. Written informed consent was obtained before commencement of interviews. The study adhered to the Consolidated criteria for reporting qualitative research (COREQ).^14^

### RELEASE resources

The development of each resource involved drawing on the authors’ previous research, and the latest evidence on antidepressant use and withdrawal symptom management.^3^^,^^5^^,^^15^^–^^17^ The authors developed a prototype for each resource and sought input for all three resources from GPs during two research meetings held at the University of Queensland’s General practice clinical unit before recruiting participants.

The medicines information brochure ‘Stopping antidepressants’ is a threefold brochure providing evidence-based information about antidepressants. The brochure is designed to raise awareness and provide information about clinical guideline recommendations, withdrawal symptoms, and how to stop taking antidepressants.

The decision aid is a two-sided A4 aid designed to support informed decision making about continuing and discontinuing antidepressants. It includes a section on comparing the pros and cons of the two options (continuing or discontinuing antidepressant use) and a list of items the patient may find useful to consider when making the decision, for example, ‘I want to feel emotion again, highs and lows’ versus ‘I would rather not feel the ups and downs’. The relevance of each item can be rated by the patient on a 5-point scale which indicates whether it is a ‘reason to stop taking’ (1 point) or a ‘reason to keep taking’ (5 points) antidepressants. The decision aid was designed according to criteria specified in international standards for enhancing the quality and effectiveness of decision aids, that is, key criteria to be defined as a decision aid and criteria for reducing the risk of making a biased decision.^18^ The brochure and decision aid are provided to the patient together.

The tapering protocol provides a step- by-step guide for slow hyperbolic tapering of antidepressant drug dose to minimise withdrawal symptoms.

### Data collection

One-to-one interviews based on the think-aloud method were conducted with participants face to face in a university meeting room or via videoconferencing (as necessitated by the COVID-19 pandemic). A researcher provided a printed copy of the RELEASE resources to participants or shared an electronic version with them via a computer screen. Participants were encouraged to say their thoughts out loud while reading the resources. The interviews were conducted and analysed in accordance with previous think-aloud research^10^ following an iterative approach, moving between data collection, analysis, modifying resources and then further data collection.

Interviews were carried out by an experienced female researcher (the first author) with qualifications in psychology and health services research, and trained in conducting semi-structured interviews. Demographical data and information on the duration of antidepressant use were collected at the start of the interview. These data included the participant’s postcode, which was mapped to quintiles based on the 2016 ‘Socioeconomic indexes for areas’ data available via the Australian Bureau of Statistics website^19^ as an indicator of socioeconomic status.

During the interview, the participant shared their thoughts out loud and were then asked open-ended questions to elicit further feedback. The researcher prompted participants to say what they liked and disliked and their general impressions about each resource. Participants saw each resource for the first time during the interview. Only the participant and researcher were present during the interview. The researcher had no existing relationship with any of the participants. Participants received a supermarket e-gift card (AUD: 50) after the interview.

Interviews were audiorecorded and transcribed with online Otter AI voice recognition software then edited for accuracy and de-identified by the first and fifth authors. Transcripts were not returned to participants for comment and/or correction and the participants did not provide feedback on the study findings.

### Analysis

A deductive coding framework was developed in accordance with the two objectives of the study — acceptability and optimisation, in consultation with previous literature,^13^ and through discussion between members of the research team. Each participant utterance was read line by line and assigned to one of the codes where relevant. Three of the researchers used the coding framework to code the 14 interview transcripts, where one researcher coded the transcript, and another checked it to determine agreement. Disagreement was discussed between the researchers. Acceptability codes and the corresponding focus of each are presented in Box 1.

**Box 1.** Coding framework for acceptability

**Resource 1. Medicines information brochure**	

Code	Focus

Relevance	The text is meaningful or relatable
Self-reported learning	The text is considered informative or educational
Motivational	The text is motivational or likely to prompt contemplation for change
Design	The text is clear, the layout or images are appropriate

**Resource 2. Decision aid**	

Code	Focus

Clarifying options	Provides useful information about the pros and cons of stopping versus continuing antidepressants enabling reflection
Clarifying preferences and values	Personal considerations for stopping or continuing antidepressants are relevant and comprehensive
Usability	Easy to follow and facilitates decision making about antidepressant use

**Resource 3. Drug-specific hyperbolic tapering protocols**	

Code	Focus

Usability	Easy to follow and enables gradual reduction and cessation of antidepressants
Self-reported learning	The text is considered informative or educational

Participant expressions suggesting the resource could be improved were assigned one of five optimisation codes:

- challenging the relevance or accuracy of text;
- additional text is required;
- confusion;
- changes to text is required; and
- layout/usability needs changing.

Coded data were extracted into an Excel spreadsheet and tabulated against each resource. Interviews were analysed in a series of four tranches, which included interviews 1–4, interviews 5–7, interviews 8–11, and then interviews 12–14. Iterative modifications were made to the resources after each tranche of interviews. Four members of the research team met after each tranche and agreement was sought about whether a modification would or would not be made. Agreed actions were documented in the spreadsheet and modifications made to resources before the next interview tranche. Participants in the next tranche read and responded to the updated resources. The collaborative nature towards making decisions about modifications and the constant comparison to participant statements in earlier tranches reduced the possibility that one participant’s perspective could dictate the process of optimising the resources.

## Results

A total of 23 adults expressed an interest in participating. Of these, nine did not take part (not eligible [*n* = 2]; not contactable [*n* = 4]; no longer interested [*n* = 1]; withdrew without providing a reason after providing written consent [*n* = 2]). The remaining 14 completed the interviews. Interviews lasted between 47 minutes and 1 hour 27 minutes (average interview length was 1 hour 1 minute). Characteristics of the participants are shown in Table 1.

**Table 1.** Participant characteristics, *N* = 14

**Characteristic**	***n* (%)**
**Sex**	
Male	2 (14.3)
Female	12 (85.7)

**Age range, years**	
18–35	4 (28.6)
36–50	6 (42.9)
51–69	3 (21.4)
≥70	1 (7.1)

**Current relationship status**	
Married/living with partner	10 (71.4)
Single/divorced/separated	4 (28.6)

**Employment status**	
Currently employed	11 (78.6)
Retired	3 (21.4)

**Socioeconomic index quintile^a^**	
1	0 (0)
2	1 (7.1)
3	0 (0)
4	3 (21.4)
5	10 (71.4)

**Medication status**	
Current taking antidepressants	11 (78.6)
Previously taking antidepressants	3 (21.4)

**Antidepressant type**	
Venlafaxine	4 (28.6)
Fluoxetine	3 (21.4)
Escitalopram	3 (21.4)
Desvenlafaxine	1 (7.1)
Duloxetine	1 (7.1)
Paroxetine	1 (7.1)
Sertraline	1 (7.1)

a *Data for socioeconomic index quintiles obtained from the Australian Bureau of Statistics.* *^19^*

Results are presented for each resource with a summary of participant views about acceptability followed by a summary of comments that led to changes to optimise the resource, with illustrative quotes throughout.

### Medicines information brochure

#### Acceptability

a) Relevance: participants reported that the brochure was fit for purpose and reflected their reality. Most participants reported experiencing the listed adverse effects or withdrawal symptoms and agreed with the reasons provided about why people keep taking antidepressants, such as fear of relapse:

*‘The first five* [adverse effects] *are all a part of my reality.’*

(Patient [P] 09, female[F]

Several participants expressed frustration at not having information on adverse effects, clinical guideline recommendation and withdrawal symptoms earlier. One participant reported that the information could have been life- changing for them:

*‘Had I had that impression, I could have done something, my life would have been quite different* […] *I have suffered “weight gain, low sex drive, inability to achieve orgasm, emotional numbing, fatigue, lack of motivation and sleep disturbance*” *for the last 18 years. I only needed to go through that for 1 year. I wish I had that information* […] *I would have not been on* [antidepressants] *for so long unnecessarily.’*

(P08, F)

b) Self-reported learning: participants were unaware that antidepressant use is so common, and some were surprised to learn that antidepressants were recommended for only 6–12 months and not for life:

*‘I’m blown away by the one in seven* [1 in 7 people in Australia] *I sometimes feel like I’m the only one I know* […] *I just thought once you’re on them, you’re on them forever.’*

(P10, F)

*‘I wish I’d known that* […] *especially the 6 to 12 months being a therapeutic guideline* […] *I would have stopped even more gradually and suffered less withdrawal symptoms if I had this information.’*

(P08, F)

Many participants did not know that withdrawal symptoms are commonly experienced when stopping antidepressants. Some misconstrued them as relapse:

*‘It hadn’t really occurred to me to think about the difference between the withdrawal symptoms and relapse. So that’s useful information.’*

(P06, F)

c) Motivational: the brochure prompted participants to reflect on their antidepressant use, with many reporting it could act as an impetus to consider stopping antidepressants:

*‘Knowing especially the 6 to 12 months* [guideline] […] *has made me more aware that perhaps it’s time to start not taking them* […] *I think this information is definitely getting me thinking about what and when I do actually start doing that.’*

(P06, F)

d) Design: participants found the brochure clear and easy to understand, particularly the language used:

*‘The language is simplistic, and anyone can understand.’*

(P05, F)

#### Optimisation

Participants challenged the accuracy of two statements in an early version of the brochure: the statements that *‘long-term antidepressant use is usually not needed’* and *‘many people incorrectly believe depression to be a lifelong condition caused by a chemical imbalance in the brain’.*

In response, both statements were modified. The first to *‘long-term use is not recommended for most people’*, and the second to: *‘many people believe depression to be a life-long condition caused by a chemical imbalance in the brain requiring lifelong treatment — which is now known to be incorrect.’*

*‘To say* “*long-term antidepressant use is usually not needed *” […] *Some people could take offence* […] *sort of say “well, hang on, you don’t know my circumstances.”’*(P04, male [M])

*‘The first dot point* [bullet point] *kind of feels a little bit condescending* […] *Like “ *incorrectly believe depression to be a lifelong condition caused by serotonin deficiency requiring lifelong treatment”*, I think that’s quite a widespread belief* […] *It makes you feel a bit stupid for believing that, if that’s not correct.’* (P14, F)

Some participants were uncertain about how they would attempt to gradually reduce the antidepressant drug dose, given mini doses are not available. In response, information about a chemist needing to compound mini-doses capsules was inserted into the brochure in addition to being already available in the tapering protocol:

*‘You can’t simply halve it nicely, to sort of cut them down, which would be nice.’*

(P03, F)

In an earlier version of the brochure participants found the term ‘hyperbolic tapering’ difficult to understand. In response, hyperbolic tapering was defined and a visual graph depicting hyperbolic tapering was added:

*‘So, you’re saying if you just decrease a small amount at a time instead of going half and half and half?* […] *A layperson statement in that way would be great because I think that people would struggle with “ *brain occupancy [refers to brain receptors and occupancy].*”’*

(P09, F).

Participants suggested improvements regarding the design of the brochure, including revising the title to make it obvious that the brochure was about stopping antidepressants (as opposed to starting) and changing the image on the front page:

*‘The picture looks a little bit desperate* […] *It kind of gives me a negative vibe, that this is like a hard decision for her to put that pill in her mouth* […] *It’s not positive.’*

(P11, F)

### Decision aid

#### Acceptability

a) Clarifying options: participants found the information about the pros and cons of stopping and continuing antidepressants to be clearly set out in the decision aid and believed this would assist people in understanding their options. Others commented the pros and cons were well balanced:

*‘I like that you’ve got them* [pros and cons] *side by side to compare, that’s really helpful and it helps to order thoughts* […] *It does come across as really balanced. It doesn’t feel like it’s trying to say you should stop taking them or you shouldn’t stop taking them.’*

(P05, F)

b) Clarifying preferences and values: the decision aid was considered thought provoking, and several participants commented that the list of factors to consider when making the decision to stop or continue antidepressants was comprehensive and helpful:

*‘If you just ask someone what matters, what are your concerns about coming off, they might give you five things, but they might forget one or two of these, so having them all there, I think it’s very good.’*

(P01, M)

The scale helped participants visualise where they are in terms of thinking about the reasons to stop or keep taking antidepressants. Participants believed the action of ticking a box on the scale for each item in the list, that is, at the ‘reason to stop’ or ‘reason to continue’ side, helped to clearly elucidate whether they were leaning towards stopping or continuing antidepressants:

*‘ Because you’ve got the boxes there, it does* […] *make it look like you’re required to do something. So that might be a good thing for some people because that sort of* […] *will prompt you to look at it further.’*

(P06, F)

c) Usability: the active process of completing the decision aid was considered helpful for prompting people to reflect on their antidepressant use and for informing their decision about whether to continue or stop:

*‘ I find this sort of thing helpful because it’s written in front of you* [...] *When it’s on the paper, it’s more, gives you clarity of where you are at* […] *you write down the pluses and minuses* […] *you kind of see more than just thinking about it in your head.’*

(P11, F)

One participant felt the decision aid was empowering in that it reinforced that they are ultimately in control of decisions about what medications to take. Others believed it would help people in communicating about their antidepressant use with their GP:

*‘ And if you show this to your GP, for example, they can then, gain a lot of information from that as well and you don’t have to try and put all your thoughts into words as well. So, I think it’s quite helpful. ’*

(P13, F)

The decision aid was described as clearly laid out and easy to follow:

*‘ It’s not easy necessarily to weigh the pros and cons* […] *I think that’s a really good layout as far as trying to get possibly what’s in someone’s head on paper.’*

(P03, F)

#### Optimisation

In an early version of the decision aid, some participants reported the information about pros and cons could be more balanced. In response, further information was provided to ensure the content and length of text was similar for both options:

*‘If you were just to pick this up and look at it* “continue taking” *wins, because that’s going to be easier. Even though when you actually read it, obviously, the detail in there is more important.’*

(P06, F)

A couple of participants also commented that the order of reasons to stop or continue should reflect priorities, and provided suggested changes to the wording used at each end of the scale to make the reasons more appropriate. Revisions were made accordingly:

*‘ *“* I don’t want to risk withdrawal symptoms *” *is one end of the spectrum, but the other end shouldn’t be “I’m ready to full brunt take on withdrawal symptoms”* […] *I feel like maybe if that was put more as “I need more support to manage withdrawal symptoms.” ’*

(P14, F)

Feedback from participants about how to improve the format and layout of the decision aid were also incorporated, such as using bullet points to list pros and cons of stopping and continuing antidepressants and reducing the number of boxes from seven to five.

### Drug-specific hyperbolic tapering protocol(s)

#### Acceptability

a) Usability: Many participants commented on the need for information about how to stop taking antidepressants and stressed they would like to see the protocols made widely available to both patients and health professionals. Some participants expressed frustration and despair at not having had this information earlier. One participant cried when reflecting on the impact this information could have had on their life if only they had had it earlier:

*‘ God, I wish I had had it* [tapering protocol] […] *Would have saved me a lot of distress* […] *I think it’s fabulous* […] *And I wish that every GP and psychiatrist who’s prescribing this drug had this amount of information and awareness* […] [it would] *make life so much better for people who’ve been prescribed this drug and want to come off.’*

(P08, F).

*‘ I’d be so happy for a lot of people be able to have access to this resource. It’s amazing.’*

(P09, F)

Many participants found the protocols clear and liked the easy-to-follow step-bystep instructions which took the *guesswork* out of tapering off antidepressants. Recording the date that each step was commenced was considered helpful:

*‘ I’ve got absolutely no idea how you would do it, so by having this information that you can keep referring back to* […] *the dates there. I think that makes it easier to follow along with and to know what’s coming up.’*

(P06, F)

The flexible and personalised nature of the tapering protocol was appreciated by many participants, who reported this could reduce their fear about stopping antidepressant use:

*‘It’s really good to have an individual medication* [protocol] […] *You feel more like it’s set for you rather than generic* […] *I think it takes a little bit of the fear away* […] *know*[ing] *that I can do it more gradually if I wanted to.’*

(P10, F)

b) Self-reported learning: participants reiterated that information in the tapering protocols about withdrawal symptoms and distinguishing them from relapse was crucial to their learning and understanding about what to expect when stopping antidepressants:

*‘I didn’t really know that you could get withdrawal symptoms* […] [and that] *they could be mistaken for, like it says, relapsing. So, it is very important probably to note these things because otherwise people will be going back to the drugs because* [they’re] *still sick.’*

(P12, F)

Some felt it was reassuring to be informed that withdrawal symptoms are ‘temporary’:

*‘ It’s almost like an affirmation* […] *“This is temporary, and other people have done it so I can do it too”. That’s really helpful* […] *especially when I’m sick from trying to* [stop antidepressants], *I just can’t think past the moment. So that’s going to be a very helpful statement* […] *I love that* [word] “temporary”*, in that it again reminds you that it’s not going to be like that forever.’*

(P05, F)

#### Optimisation

Earlier iterations of the tapering protocol provided information on steps the patient could take when experiencing withdrawal symptoms depending on their severity. These were considered ambiguous to a few participants and created uncertainty about what to do, particularly if severe withdrawal symptoms were experienced:

*‘ *“Mild, moderate, severe”*. That’s quite subjective* […] *I know what one person considers mild, another person probably thinks of as severe. So that’s a bit ambiguous.’*

(P14, F)

In response, modifications were made to remove the ambiguity, enabling participants to make their own assessment about the severity of withdrawal symptoms and whether to pause on the current dose or return to the previous dose.

## Discussion

### Summary

This think-aloud study among adults with lived experience of long-term antidepressant use informed optimisation of the RELEASE resources designed to support safe cessation of long-term antidepressant use in general practice. Participants found the resources acceptable, useful, comprehensible, and reassuring. The resources fulfilled their intended purpose of raising awareness and recognition of withdrawal symptoms, supporting informed decision making, and guiding safe cessation of antidepressants. Several iterative modifications were made across the three resources in response to participant feedback to address areas of ambiguity, increase objectivity, improve suitability of the language; and to enhance design, format, and layout.

### Strengths and limitations

A strength of the study was the inclusion of adults with lived experience of long-term antidepressant use to explore acceptability and inform optimisation of the resources. This ensured that the resources are relevant to their target population. Data collection and analysis were conducted in parallel, which enabled iterative testing of modifications of the resources.

A potential limitation of the study was the small sample size of 14 participants, only two males and few older people. However, the issues raised about each resource were similar across participants. While the study included a representative sample of participants in relation to age and sex, people with low socioeconomic status were under-represented which could limit the applicability of findings.

Several participants demonstrated a motivation to stop taking antidepressants, which could have led to exaggerated accounts of acceptability and usability of the resources. The use of pre-defined codes to analyse interview data can limit the identification of emergent themes. While this may have occurred, the focus of the interviews was to elicit immediate real-time feedback with participants engaging with the resources rather than to explore in-depth perspectives about antidepressants.

### Comparison with existing literature

Supporting patients to stop long-term antidepressant use by focusing on the physiological aspects associated with antidepressant cessation is receiving increased recognition,^1^^,^^3^^,^^4^^,^^20^^,^^21^ and is central to RELEASE. While much previous research included psychological support for stopping antidepressants,^22^^–^^24^ RELEASE focuses on raising awareness and recognition of withdrawal symptoms and providing step- by-step guidance on tapering to minimise withdrawal symptoms. This approach resonated with the study participants, supporting findings from previous qualitative research identifying withdrawal symptoms as a key barrier to stopping antidepressants.^5^^,^^25^

The RELEASE resources were optimised based on input from individuals with lived experience, which is known to improve uptake.^10^ Patient engagement during intervention development is crucial to intervention effectiveness to solve real- world issues.^26^

### Implications for research and practice

This think-aloud study among adults with lived-experience of long-term antidepressant use assessed the acceptability and informed optimisation of the RELEASE resources designed to support safe cessation of long-term antidepressants in general practice.

A cluster randomised controlled trial is ongoing to test the effectiveness of these resources as part of the RELEASE multistrategy intervention in general practice.
